# The Role of Radiotherapy to the Primary Site in Oropharyngeal Cancer with Limited Metastases—An Analysis of a Hospital-Based Registry

**DOI:** 10.3390/cancers16244130

**Published:** 2024-12-11

**Authors:** Michael Kharouta, F. Jeffrey Lorenz, Sean Mahase, Hongyun Shi, Neerav Goyal, Min Yao

**Affiliations:** 1 Department of Radiation Oncology, Creticos Cancer Center, Advocate Illinois Masonic Medical Center, Chicago, IL 60657, USA; 2 Department of Otolaryngology–Head and Neck Surgery, Penn State College of Medicine, Hershey, PA 17033, USA; 3 Department of Radiation Oncology, Penn State College of Medicine, Hershey, PA 17033, USA; 4 Department of Radiation Oncology, Affiliated Hospital of Hebei University, Baoding 071000, China; hyshi2015@163.com

**Keywords:** oligometastasis, oropharyngeal cancer, radiation, head and neck cancer

## Abstract

**Background/Objectives:** Limited metastatic squamous cell carcinoma of the oropharynx (OPC) lacks clear management guidelines, especially for HPV-associated disease. The objective of this study was to investigate if primary site radiotherapy (RT) benefits overall survival in limited metastatic OPC. **Methods:** Utilizing the National Cancer Database (NCDB), patients aged 18–90 with OPC presenting as cM1 with limited metastatic disease to one distant site were identified. Propensity score matching, Cox-proportional hazards models, and Kaplan–Meier estimates were employed to assess factors associated with overall survival. **Results:** In this study, 1056 patients were included with metastases involving bone (19.0%), brain (0.8%), lung (52.9%), liver (10.1%), and lymph nodes (20.4%). Treatment modalities included 54.6% receiving primary site RT, 45.4% receiving no RT, and 69.9% undergoing systemic therapy. For HPV-positive patients, RT (HR 0.64, *p* = 0.0026) and receipt of chemotherapy (HR = 0.57, *p* = 0.0057) were associated with improved overall survival, while bone and lung metastases were associated with decreased survival (HR = 1.75 and 1.39, *p* = 0.0041 and 0.041, respectively). In HPV-negative cases, survival also correlated with RT (HR = 0.65, *p* = 0.0047), receipt of chemotherapy (HR = 0.45, *p* < 0.001), clinical T4 disease (HR = 1.99, *p* = 0.012), presence of bone metastases (HR = 2.52, *p* < 0.001), lung metastases (HR = 1.49, *p* = 0.035), and lymphovascular invasion (HR = 1.10, *p* < 0.001). Overall, patients who received RT showed increased median overall survival from 9.9 to 16.1 months (*p* < 0.001) compared to those who did not. When stratified by RT and HPV status, there was higher median survival for both HPV-positive (from 17.1 to 24.9 months, *p* < 0.001) and HPV-negative patients (from 8.4 to 12.9 months, *p* = 0.0016) who received RT compared to those who did not. **Conclusions:** Primary-site radiotherapy may positively impact overall survival in limited metastatic OPC, irrespective of HPV status.

## Simple Summary

This study explores whether radiation therapy at the primary tumor site improves survival in patients with oropharyngeal cancer with distant metastasis limited to one anatomic site. We used data from a large national database to examine over 1000 patients, many of whom also received systemic therapy. Our findings suggest that radiation treatment to the primary cancer site can extend survival in patients, regardless of human papillomavirus status. This research may help guide treatment options for patients with limited metastatic oropharyngeal cancer, potentially improving survival outcomes in this group.

## 1. Introduction

Oropharyngeal cancer (OPC) primarily includes cancers of the tonsils and base of the tongue. Treatment varies by stage: early or locally advanced disease is typically managed with surgery, radiation therapy (RT), and/or chemotherapy with curative intent [1]. Although early-stage OPC is associated with a relatively favorable prognosis, the presence of distant metastasis is linked to poor outcomes [2]. The overall incidence of metastatic disease at presentation is low—estimated at 3–10% for all head and neck squamous cell carcinoma (HNSCC) subsites [3,4]. In the past, chemotherapy was the cornerstone of systemic treatment for metastatic disease, often given with palliative intent. In recent years, treatment approaches have progressed due to advances in immunotherapy, and treatment typically entails chemotherapy, immunotherapy, and, in selected cases, RT [5,6]. Despite advances in treatment, OPC with distant metastasis remains difficult to treat, with low survival rates estimated to be 40.8% at two years [7].

The optimal treatment strategy for patients with limited metastasis—defined here as having a single distant metastatic site and considered an intermediate disease state—remains unclear and is an area of ongoing research [8]. In recent years, the role of targeted local therapy, such as RT administered to the primary tumor site, has emerged as a potential treatment option. There is limited existing data on this topic, but retrospective studies have demonstrated an association between locoregional RT and improved survival in this setting [9]. This approach is based on the understanding that progression of the primary tumor can lead to increased bleeding, respiratory obstruction, and cachexia [10], and that most patients with distant metastatic HNSCC ultimately succumb to the disease due to ongoing progression of the primary tumor and locoregional disease [11,12]. As such, targeting the primary tumor may enhance survival outcomes by reducing tumor burden, even in the presence of metastatic disease. The survival advantage may also arise from reduced risk of further tumor spread or seeding [13] and reduced circulating tumor mediators such as growth factors and cytokines [14].

Moreover, the role of human papillomavirus (HPV) status in determining which patients are most likely to benefit from primary site RT is of particular interest. HPV-positive OPC is now more common, and it differs in epidemiologic predilection, with distinct clinical and molecular features [15]. HPV-positive OPC is more likely to affect younger, non-smoking individuals, while HPV-negative disease is associated with a history of smoking and alcohol. HPV-positive cancers are more radiosensitive and responsive to treatment [16,17]. The 3-year overall survival is reported to be 82.4% for HPV-positive disease and 57.1% in HPV-negative disease [18]. As a result, there are separate staging systems for these cancers [19].

The goal of this study was to elucidate the clinicopathologic factors associated with overall survival in patients with OPC and limited metastatic disease at presentation. Additionally, we aimed to determine whether RT to the primary site confers a survival benefit and if this benefit is differentially dependent on the HPV status of the tumor.

## 2. Materials and Methods

This study utilized data from the National Cancer Database (NCDB). The NCDB is a comprehensive and nationwide repository that captures information from more than 1500 cancer programs accredited by the Commission on Cancer (CoC) of the American College of Surgeons, which represents approximately 70% of cancers diagnosed in the United States [20]. The NCDB provides a wealth of clinical, pathological, and demographic data, allowing for a robust analysis of cancer-related outcomes. The NCDB data are de-identified, publicly available, and do not involve direct interaction with patients or animals. Therefore, the Pennsylvania State University Institutional Review Board (IRB) review deemed STUDY00018678 as exempt from review on 6 October 2021, and informed consent was not applicable. The use of this dataset is in compliance with regulations governing secondary data analysis.

**Figure 1:**
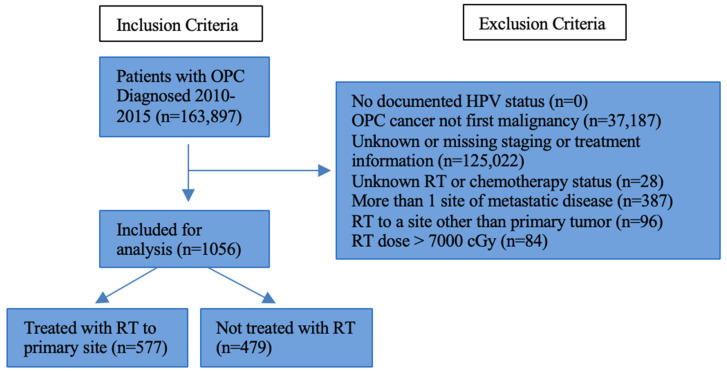
Inclusion and exclusion criteria. Abbreviations: OPC, oropharyngeal cancer; RT, radiotherapy; HPV, human papillomavirus; cGy, centigray.

The NCDB was queried from 2010 to 2015 for patients aged 18–90 years with OPC of any subsite (which included oropharynx, tonsil, and base of tongue) categorized as cM1 with limited metastatic disease, which was defined as metastatic disease to only one distant site. In the NCDB, patients are coded for what other organ sites have metastatic spread of disease (bone, liver, lung, brain, and/or lymph nodes). Patients were defined as having “limited metastatic disease” if they had only one of these sites with metastatic disease. We inferred from the data provided in the NCDB that patients with cM1 disease and with the only known site of metastasis being “lymph node” that these must represent spread to distant lymph nodes, such as mediastinal and/or axillary nodes. This definition was chosen for the most favorable subset of metastatic patients within the confines of the data dictionary of the NCDB, which does not provide the number of metastatic lesions or volume of metastatic disease. Included subjects must have a documented HPV status and first-course RT to the primary site or no RT. Patients for whom oropharynx cancer was not their first primary malignancy (n = 37,187), with unknown or missing staging or treatment data (n = 125,022), with unknown radiotherapy or chemotherapy status (n = 28), with more than one site of metastatic disease (n = 387), receiving radiotherapy to a site other than the primary site (n = 96), and with radiation doses in excess of 7000 cGy (n = 84) were excluded from the study. A flow chart detailing inclusion and exclusion criteria is presented in Figure 1.

As this is a population-based observational study, patients were not randomized to treatment groups, which introduces the potential for selection bias and confounding variables influencing the outcomes. To address this, full-optimal propensity score matching (PSM) was employed to create a balanced cohort. PSM allowed for the adjustment of observed baseline covariates by matching patients receiving different treatments but with similar propensity scores, which are estimated probabilities of receiving the treatment given their covariates. This method enhanced causal inference by reducing confounding. The assumption with PSM was that all relevant confounders that affect both treatment assignment and outcome are observed and included in the model. To achieve this, a comprehensive set of covariates known or suspected to influence both the likelihood of receiving radiotherapy and survival outcomes was included. Full-optimal PSM was performed for the propensity of receipt of RT based on clinicopathologic covariates, which included age, sex, race, Charlson–Deyo comorbidity index, tumor grade, HPV status, presence of metastases in bone, brain, lung, liver, and lymph nodes, tumor size (in cm), presence of lymphovascular invasion (LVSI), clinical T category, clinical N category, and receipt of any systemic therapy or immunotherapy. Covariate balance after matching was assessed graphically using a Love plot comparing pre- and post-matching absolute standardized mean differences, which shows significantly improved balance between covariates after matching. For the Love plot standardized mean difference threshold, 0.1 was selected to indicate that covariates were balanced because this is among the more stringent of the commonly used thresholds and represents a negligible difference.

Using the propensity-weighted cohort, univariate and multivariate Cox-proportional hazards (CPH) models were generated using bi-directional stepwise regression to identify covariates associated with overall survival. The Akaike information criterion (AIC) was calculated for each model, and the model minimizing the AIC was selected. For the CPH model, there was the assumption of proportional hazards between groups. Scaled Schoenfeld residuals were generated to assess the validity of the proportional hazards assumption for the selected models, which, for the whole cohort, showed that proportional hazards assumption was violated. Stratification by HPV status corrected this violation, allowing the baseline hazard function to vary across these strata by HPV status. Kaplan–Meier estimates of overall survival were generated and stratified by receipt of RT to the primary site and HPV status. Overall survival was compared between these groups using log-rank testing. Because the multivariate model for the overall cohort violated the proportional hazards assumption, CPH models stratified by HPV status were generated. All statistical analyses were conducted using the R open source language and environment for statistical computing (version 4.4.1, Vienna, Austria) with 2-sided tests and a significance level of *p* = 0.05. This *p* value cutoff was selected as it is a common accepted convention in biomedical statistics. This threshold indicates that there is less than a 5% probability that the observed effect occurred by chance under the null hypothesis, which many consider a small enough probability of error.

## 3. Results

### 3.1. Cohort Demographics and Clinical Characteristics

A total of 1056 patients with OPC diagnosed between 2010 and 2015 met inclusion criteria for this study. Cohort demographic characteristics are summarized in Table 1. The median age for all patients was 61 years, and 81.0% of patients were White, 15.4% Black, and 3.6% other race or unknown.

Cohort disease and clinical characteristics are presented in Table 2. Regarding the disease subsite, 40.4% were classified as the tonsil, 41.2% as the base of the tongue, and 18.4% as another oropharynx tissue. The median follow-up was 12.4 months for all patients and 43.9 months for surviving patients. By clinical T category, 11.1% of patients were cT1, 27.9% were cT2, 24.1% were cT3, and 36.8% were cT4. By the clinical N category, 5.8% were cN0, 9.9% were cN1, 70.2% were cN2, and 14.1% were cN3. By the site of metastasis, 19.0% of patients had bone metastases, 0.8% had brain metastases, 52.9% had lung metastases, 10.1% had liver metastases, and 20.4% had lymph node metastases. There were 54.6% of patients who received RT to the primary site, 45.4% of patients received no RT, and 69.9% received some form of systemic therapy. Of the patients receiving RT, 58.4% received intensity modulated RT, 36.4% received external beam not otherwise specified, and 3.8% received 3D conformal RT, with several patients (1.4%) receiving other techniques including 2D RT, stereotactic body RT, or unknown. Less than 10% of patients were coded as having received immunotherapy. In terms of surgery, approximately 90% of patients had no surgery. The remaining 10% had surgeries of various scopes at the base of tongue or tonsil, or it was unknown if they had surgery, with 7.4% of all patients receiving postoperative RT, 2% unknown, and less than 1% receiving split course RT before and after surgery.

### 3.2. Clinical Characteristics Associated with Survival in Limited Metastatic OPC

**Figure 2:**
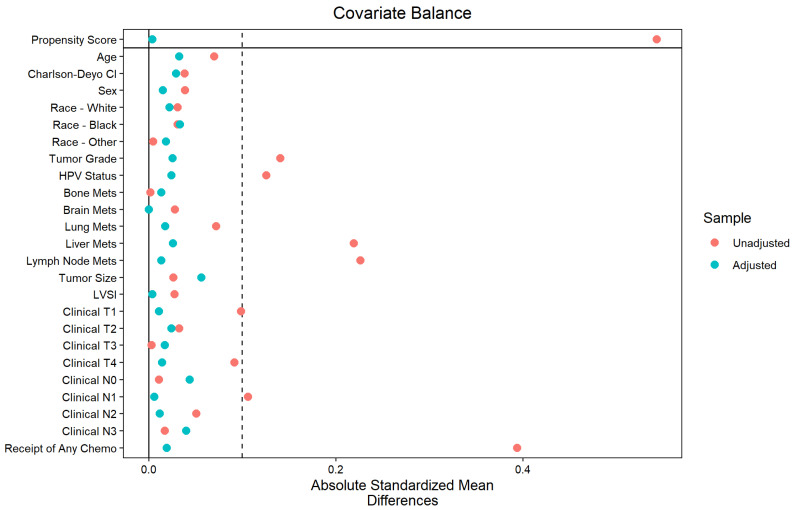
Love plot of covariate balance in the entire cohort before and after matching for propensity of receipt of radiotherapy. There was enhancement in balance across nearly all variables, with improvements falling below the conventional threshold of 0.1 for absolute standardized mean differences.

The assessment of the Love plot generated from the matched data showed balance improvement in all variables below the conventional absolute standardized mean difference threshold of 0.1 (Figure 2). After matching, a CPH model was generated using stepwise regression for association of covariates with overall survival (Table 3). CPH regression using clinicopathologic covariates violated the proportional hazards assumption. This was mitigated by stratifying patients by HPV status.

The results of CPH modeling after stratification are shown in Table 4. Multivariable CPH regression showed that RT was associated with a benefit to overall survival regardless of HPV status. For HPV-positive patients, RT (HR 0.64, *p* = 0.0026) and receipt of chemotherapy (HR = 0.57, *p* = 0.0057) were associated with improved overall survival, while bone and lung metastases were associated with decreased survival (HR = 1.75 and 1.39, *p* = 0.0041 and 0.041, respectively). In HPV-negative cases, survival also correlated with RT (HR = 0.65, *p* = 0.0047), receipt of chemotherapy (HR = 0.45, *p* < 0.001), clinical T4 disease (HR = 1.99, *p* = 0.012), presence of bone metastases (HR = 2.52, *p* < 0.001), lung metastases (HR = 1.49, *p* = 0.035), and lymphovascular invasion (HR = 1.10, *p* < 0.001). There was an association with the presence of brain metastases and survival for both HPV-positive and negative patients; however, only eight patients presented with this site of metastasis, and thus these associations were not meaningfully interpretable in our model as evidenced by the extreme hazard ratios.

### 3.3. Kaplan–Meier Survival Analysis Stratified by RT to the Primary Site and HPV Status

**Figure 3:**
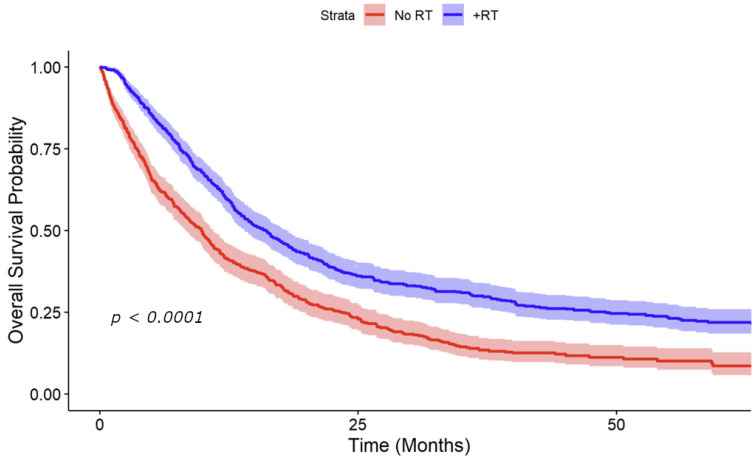
Kaplan–Meier curve adjusted for propensity-weighted data stratified by receipt of radiotherapy. These curves revealed a notable improvement in median overall survival, rising from 9.9 to 16.1 months with the administration of RT (*p* < 0.001). A 95% confidence interval is shown.

**Figure 4:**
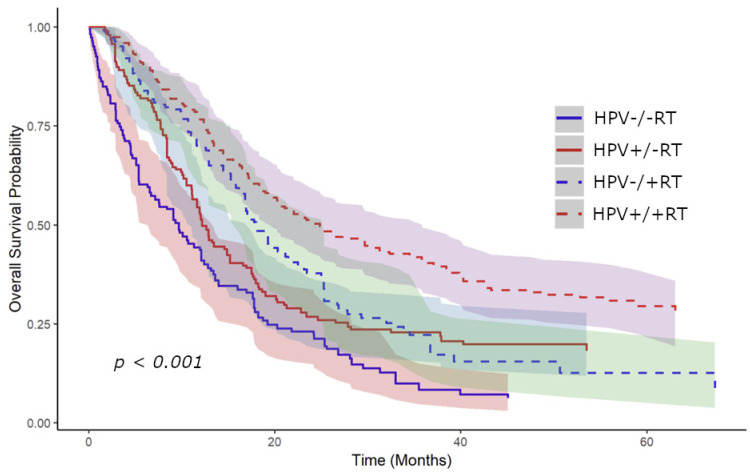
Kaplan–Meier curve adjusted for propensity-weighted data stratified by receipt of radiotherapy and HPV status. There was a rise in median survival for HPV-positive patients from 17.1 to 24.9 months with the receipt of RT (*p* < 0.001) and for HPV-negative patients from 8.4 to 12.9 months (*p* = 0.0016). A 95% confidence interval is shown for all curves.

Kaplan–Meier curves were generated for overall survival using the propensity-weighted data. These curves were initially stratified by receipt of RT to the primary site, showing an improvement in median overall survival from 9.9 to 16.1 months with receipt of RT (*p* < 0.001) (Figure 3). Stratified curves by both RT and HPV status were subsequently generated showing improvement in median survival with receipt of RT for HPV-positive patients from 17.1 to 24.9 months (*p* < 0.001) and from 8.4 to 12.9 months (*p* = 0.0016) for HPV-negative patients (Figure 4). Patients with unknown HPV status also showed an improvement in median survival from 7.9 to 12.9 months (*p* < 0.001). 

## 4. Discussion

This study provides valuable insights into managing OPC with limited metastasis, particularly regarding the role of RT to the primary site and the influence of HPV status on overall survival. Analyzing a large patient cohort, we found that RT improves survival outcomes for all patients regardless of HPV status, even after adjusting for chemotherapy. RT to the primary site in the setting of metastatic disease is not unique to HNSCC or OPC. The principles have been demonstrated to extend to other cancers as well, including lung [21], prostate [22], urothelial [23], and breast cancer [24]. It is intuitive that patients with a lower metastatic disease burden, especially relative to their primary disease site, may derive more benefit from definitive local therapy. This benefit is likely due to improved local control of the primary tumor, the prevention of invasion into vital organs in the oropharynx, a lower risk of tumor spread or seeding [13], and a decrease in circulating tumor mediators like growth factors and cytokines [14].

In this study, we defined a cohort of patients with a possible lower burden of metastatic disease limited to a single organ since NCDB does not provide information regarding the number of metastases. The survival benefit from RT to the primary site supports the recent literature which advocates for more aggressive local treatment in metastatic disease [9]. In a study of 65 patients with metastatic HNSCC by Rambeau et al., overall survival was 16.5 months for patients who underwent locoregional RT compared to 7.5 months for those who did not [25]. Nguy et al. analyzed the NCDB from 2004 to 2015 and found that, in a cohort of 556 patients with metastatic OPC and known HPV status, radiotherapy was significantly associated with improved 1-year overall survival (67% vs. 58%) [26]. However, in contrast to the current study, when stratified by HPV status, head and neck RT conferred a survival advantage only for patients with HPV-positive disease. Several factors may explain this difference. Notably, the previous study did not restrict its analysis to patients with limited metastatic disease, with nearly 40% of patients having multiple metastatic sites. It is possible that HPV-negative disease with multiple metastatic sites represents a more aggressive disease state, while those with limited metastasis may have a more favorable prognosis. Furthermore, there was a trend towards improved survival in the HPV-negative group treated with RT even though it was not statistically significant (hazard ratio 0.83, 95% CI 0.63–1.10, *p* = 0.194), suggesting that the study may have lacked sufficient statistical power to detect a difference. The inclusion of data as early as 2004 in Nguy et al.‘s study may have also influenced the results. In another study of 3516 patients with metastatic HNSCC by Kabarriti et al., the addition of RT to chemotherapy was associated with prolonged overall survival, with a median survival of 13.6 months compared to 11.3 months for those who did not receive RT [27]. Zumsteg et al. analyzed the NCDB and found that high-intensity local therapy (>60 Gy radiotherapy or oncologic resection) combined with systemic therapy improved survival in metastatic HNSCC, while low-intensity local treatment yielded results similar to systemic therapy alone [28]. Another small study of seven HNSCC patients with synchronous distant metastases found that those treated with aggressive radiotherapy had significantly better survival (14 months vs. 5.5 months) and 60-day mortality (0% vs. 50%) [29]. You et al. reported significant improvement in two-year OS from 55% to 76% for patients with de novo metastatic nasopharyngeal carcinoma undergoing definitive locoregional therapy after induction chemotherapy. However, this study highlighted the need for careful patient selection, reporting rates of grade 3 or 4 deafness (5%), trismus (3%), and mucositis (34%) in those receiving definitive therapy [30]. Together, these results underscore the potential benefits of primary site treatment in selected patients with metastatic head and neck cancers, warranting further prospective evaluation.

In this study, HPV-positive OPC had improved overall survival compared to HPV-negative, consistent with the existing literature [31]. Both groups experienced a survival benefit from primary site RT. Specifically, HPV-positive patients showed a median survival increase from 17.1 to 24.9 months, while the survival of HPV-negative patients improved from 8.4 to 12.9 months. Those with HPV-positive disease had larger benefits in terms of the gross number of months; however, the proportional increases were comparable. Although HPV-positive tumors are generally considered more radiosensitive due to impaired DNA repair mechanisms and augmented immune response [32,33], these findings indicate that RT to the primary site may offer significant benefits for all patients with limited metastatic OPC, regardless of HPV status.

Our study also highlights the critical role of systemic therapy, particularly chemotherapy, in improving overall survival across both HPV status groups. This finding supports current treatment paradigms that advocate for multimodal approaches, combining local and systemic therapies to achieve optimal outcomes in patients with advanced disease [1,34]. Chemotherapy is commonly combined with radiotherapy in the setting of OPC to synergistically boost the effectiveness of treatment [35]. Additionally, the integration of immunotherapy into treatment regimens has shown promise in further improving survival for patients with recurrent or metastatic disease [36]. Borson et al. conducted a retrospective study of 40 patients with metastatic HNSCC treated with definitive surgery or chemoradiation directed at the primary site. The median OS was 14.2 months, which increased to 27.5 months with induction therapy and nearly 42 months with anti-PD1 therapy. This emphasized the therapeutic potential of treatment directed at the primary site, especially when combined with induction and/or immunotherapy [37].

It is noteworthy that despite the survival benefit associated with RT, patients with certain clinical features, such as clinical T4 disease, bone metastases, or lung metastases, demonstrated poorer prognoses. A prior study of 40 patients showed that among patients with oligometastatic disease treated with radical local therapy, improved survival was significantly linked to better ECOG performance status, the absence of bone and brain metastases, and a smaller total tumor volume [38]. A significant concern with definitive treatment for HNSCC patients with metastatic disease at presentation is the considerable toxicity of therapy [39]. Improper patient selection can lead to an unfavorable therapeutic ratio, reducing the likelihood of quality of life or survival benefits [40]. These findings highlight the need for careful patient selection and treatment planning through a multidisciplinary approach.

Our study has a number of limitations, apart from the obvious limitation of its retrospective and observational nature. First, while NCDB allows for the determination of the number of metastatic sites, it does not provide the total number of metastatic lesions. It is also not possible to account for whether radiation was also directed at metastatic lesions, which is another treatment approach gaining popularity in the oligometastatic setting [38,41]. It should be noted that the NCDB lacks critical prognostic factors for HNSCC, such as smoking status, and it only reports overall survival outcomes [42]. We attempted to reduce selection bias through propensity-score matching and multivariate adjustment for factors like age and comorbidity score, but these methods cannot replace a randomized, prospective study design. It was also not possible to determine any specifics of the RT protocol including dose, fractionation, whether the course was completed, and the intent of therapy. Finally, the recent introduction of immunotherapy in HNSCC treatment impacts survival [43,44], but less than 10% of patients in our study received it.

## 5. Conclusions

In conclusion, our analysis shows that RT to the primary site is associated with improved overall survival in patients with OPC with single-site metastatic disease, regardless of HPV status. Currently, our clinical approach for this patient population involves initiating several cycles of chemoimmunotherapy, followed by locoregional radiation to the head and neck if no disease progression is observed. In the absence of randomized data, we have reserved treatment of the primary site for those with the best prognostic factors. For example, delivering treatment to the primary site should be considered for HPV-positive patients with limited metastatic disease, including a solitary lesion, excellent performance status, younger age, and a low burden of regional nodal disease. However, our data demonstrate that patients with HPV-negative disease may also derive benefit and be candidates for this treatment approach. The treatment plan for this patient population should be made on a patient-by-patient basis, and they should be discussed with a multidisciplinary team and be considered for clinical trials. Given the promising role of RT, further studies are needed to better understand which patient characteristics predict the most favorable outcomes to refine patient selection criteria. Future prospective trials should validate these findings and investigate the optimal combination of RT with systemic therapies, including immunotherapy, to improve survival in this challenging patient group.

## Figures and Tables

**Table 1:** Cohort demographics.

Patient Variable	Total Patients, n (%) (n = 1056)
**Median Age (range)**	61 (30–90)
**Sex**	
Male	877 (83.0%)
Female	179 (17.0%)
**Race**	
White	855 (81.0%)
Black	163 (15.4%)
Other/Unknown	38 (3.6%)

**Table 2:** Cohort clinical and disease characteristics.

Patient Variable	Total Patients, n (%) (n = 1056)
**Charlson–Deyo Comorbidity Score**	
0	797 (75.5%)
1	177 (16.8%)
2	57 (5.4%)
3	25 (2.4%)
**Tumor Subsite**	
Tonsil	427 (40.4%)
Base of Tongue	435 (41.2%)
Other Oropharynx	194 (18.4%)
**Tumor Grade**	
1	36 (3.4%)
2	257 (24.3%)
3	326 (30.9%)
4	11 (1.0%)
Unknown	426 (40.3%)
**HPV Status**	
Positive	217 (20.5%)
Negative	280 (26.5%)
Unknown	559 (52.9%)
**Clinical T Stage**	
cT1	117 (11.1%)
cT2	295 (27.9%)
cT3	255 (24.1%)
cT4	389 (36.8%)
**Clinical N Stage**	
cN0	61 (5.8%)
cN1	105 (9.9%)
cN2	741 (70.2%)
cN3	149 (14.1%)
**Site of Metastasis**	
Bone	201 (19.0%)
Brain	8 (0.8%)
Lung	559 (52.9%)
Liver	107 (10.1%)
Distant Lymph Node	215 (20.4%)
**Presence of Lymphovascular Invasion**	
Yes	168 (15.9%)
No	66 (6.3%)
Unknown	822 (77.8%)
**Radiotherapy**	
Yes	577 (54.6%)
No	479 (45.4%)
**Systemic Therapy**	
Yes	738 (69.9%)
No	318 (30.1%)
**Immunotherapy**	
Yes	89 (8.5%)
No	967 (91.5%)

Abbreviations: HPV, Human papillomavirus.

**Table 3:** Cox proportional hazards model for association of covariates with overall survival.

	Univariate		Multivariate	
	HR	*p*-Value	HR	*p*-Value
**Any RT**	0.72	<0.001	0.7	<0.001
**HPV Status**	1.03	0.0017	1.02	0.049
**Age**	1.02	<0.001	1.01	0.0024
**CDCC**	1.21	0.0011	1.16	0.0021
**T Category**				
1	-	-	-	-
2	1.27	0.06	1.13	0.36
3	1.38	0.027	1.35	0.025
4	1.8	<0.001	1.64	<0.001
**N Category**				
0	-	-	-	-
1	1.16	0.44	1.34	0.11
2	1	0.99	1.28	0.11
3	1.24	0.24	1.53	0.014
**Tumor Size**	1	0.84	-	-
**Bone Mets**	1.34	0.0018	1.81	<0.001
**Liver Mets**	1.11	0.44	1.41	0.0045
**Lung Mets**	1.35	<0.001	1.58	<0.001
**Brain Mets**	1.74	0.087	2.13	0.069
**LN Mets**	0.54	<0.001	-	-
**LVSI**	1.02	0.056	1.02	0.04
**Any Chemo**	0.5	<0.001	0.55	<0.001

Abbreviations: HR, Hazard ratio; RT, Radiotherapy; HPV, Human papillomavirus; CDCC, Charlson–Deyo comorbidity index; Mets, Metastasis; LN, Distant Lymph node; LVSI, Lymphovascular invasion; Chemo, Chemotherapy.

**Table 4:** Results of Cox proportional hazards modeling after stratification.

	HPV Positive				HPV Negative			
	Univariate		Multivariate		Univariate		Multivariate	
	HR	*p*-Value	HR	*p*-Value	HR	*p*-Value	HR	*p*-Value
**Any RT**	0.67	0.013	0.64	0.0026	0.69	0.018	0.65	0.0047
**Age**	1.01	0.089	1.01	0.093	0.99	0.27	0.99	0.14
**CDCC**	1.16	0.288	1.22	0.052	1.11	0.28	1.28	0.017
**T Category**								
1	-	-	-	-	-	-	-	-
2	1.76	0.038	-	-	1.11	0.68	1.12	0.7
3	2.04	0.0088	-	-	1.35	0.26	1.27	0.42
4	1.7	0.062	-	-	2.13	0.0025	1.99	0.012
**N Category**								
0	-	-	-	-	-	-	-	-
1	0.73	0.56	-	-	1.44	0.37	-	-
2	0.73	0.49	-	-	1.31	0.44	-	-
3	0.87	0.78	-	-	1.65	0.23	-	-
**Tumor Size**	1	0.97	-	-	1	0.016	1	0.11
**Bone Mets**	1.42	0.048	1.75	0.0041	1.89	0.0027	2.52	<0.001
**Liver Mets**	1.11	0.616	-	-	0.79	0.45	-	-
**Lung Mets**	1.35	0.044	1.39	0.041	1.14	0.401	1.49	0.035
**Brain Mets**	0	*p* < 0.001	-	-	44.03	<0.001	48.53	<0.001
**LN Mets**	0.6	0.0061	-	-	0.69	0.042	-	-
**LVSI**	0.99	0.6	-	-	1.06	0.014	1.1	<0.001
**Any Chemo**	0.66	0.074	0.57	0.0057	0.59	0.0093	0.45	<0.001

Abbreviations: HR, Hazard ratio; RT, Radiotherapy; HPV, Human papillomavirus; CDCC, Charlson–Deyo comorbidity index; Mets, Metastasis; LN, Distant lymph node; LVSI, Lymphovascular invasion; Chemo, Chemotherapy.

## Data Availability

The study data are available from the corresponding author upon reasonable request.
